# Satb2 and Nr4a2 are required for the differentiation of cortical layer 6b

**DOI:** 10.1038/s41420-025-02402-2

**Published:** 2025-03-31

**Authors:** Li Zhao, Yun-Chao Tao, Ling Hu, Xi-Yue Liu, Qiong Zhang, Lei Zhang, Yu-Qiang Ding, Ning-Ning Song

**Affiliations:** 1 Laboratory Animal Center, Fudan University, Shanghai, China; 2 State Key Laboratory of Medical Neurobiology and MOE Frontiers Center for Brain Science, Institutes of Brain Science, Fudan University, Shanghai, China; 3 Shanghai Yangzhi Rehabilitation Hospital (Shanghai Sunshine Rehabilitation Center), Tongji University School of Medicine, Shanghai, China; 4 Clinical Center for Brain and Spinal Cord Research, Tongji University, Shanghai, China; 5 Shanghai Institute of Infectious Disease and Biosecurity, Fudan University, Shanghai, China; 6 Huashan Institute of Medicine (HS-IOM), Huashan Hospital, Fudan University, Shanghai, China

**Keywords:** Developmental neurogenesis, Differentiation

## Abstract

Cortical layer 6 is divided into two sublayers, and layer 6b is situated above the white matter with distinct architecture from layer 6a. Layer 6b arises from the subplate and contains the earliest born neurons in the development of cerebral cortex. Although great progress has been made in understanding the cortical morphogenesis, there is a dearth of knowledge regarding the molecular mechanisms governing the development of layer 6b neurons. Here we report that transcription factor special AT-rich binding protein 2 (Satb2) and nuclear receptor subfamily 4 group A member 2 (Nr4a2) are required for the normal differentiation layer 6b neurons. Upon conditional deletion of Satb2 in the cortex (Satb2^Emx1^ CKO) or selectively inactivation of Satb2 in layer 6b neurons only (Satb2^Nr4a2CreER^ CKO), the expressions of layer 6b-specific genes (i.e., Ctgf, Cplx3, Trh and Tnmd) were significantly reduced, whereas that of Nr4a2 was dramatically increased, underscoring that Satb2 is involved in the differentiation of layer 6b neurons in a cell-autonomous manner. On the other hand, when Nr4a2 was deleted in the cortex, the expressions of Trh and Tnmd were upregulated with unchanged expression of Ctgf and Cplx3. Notably, the defective differentiation resulting from the deletion of Satb2 remained in Satb2/Nr4a2 double CKO mice. In summary, our findings indicated that both Satb2 and Nr4a2 are required for the differentiation of layer 6b neurons possibly via different pathways.

## Introduction

Layer 6b, the deepest neocortical layer, lies along the border between gray matter and white matter. Although layer 6 is usually considered as a single uniform layer, neurons in layer 6b are distinct from those in layer 6a developmentally, genetically and morphologically [1–4]. Layer 6b arises from the subplate in the developing cerebral cortex, containing the earliest born neurons in brain [2, 3]. The expression of subplate-specific genes, including connective tissue growth factor (Ctgf), complexin 3 (Cplx3) and nuclear receptor subfamily 4 group a member 2 (Nr4a2), persists into adulthood in layer 6b [4, 5]. During development, the subplate neurons are essential for the establishment of early postnatal thalamocortical projections and control radial migration of cortical neurons via transient synapse-like contacts [5–7]. Layer 6b has been reported to be implicated in psychiatric disorders, such as autism or schizophrenia [3, 5, 6]. Additionally, layer 6b is the only layer responsive to the neuropeptide orexin, and neurons in layer 6b significantly influence the brain state [8, 9]. However, layer 6b has remained poorly characterized so far, especially regarding the genetic mechanisms underlying its development.

The transcription factor special AT-rich sequence binding protein 2 (Satb2) binds to the nuclear matrix attachment regions (MARs) within the genome and regulates gene expression as a chromatin remodeler [10, 11]. Satb2 was first identified as a gene responsible for cleft palate and Satb2 mutation in mice leads to perinatal death and severe craniofacial defects [12–14]. In cortical development, Satb2 is a determinant gene for the cell fate of callosal projection neurons [15–17] and for the regionalization of the retrosplenial cortex by suppressing Ctip2 and Nr4a2 [18]. Satb2 is also required for the dendritic self-avoidance and somal spatial arrangement of cortical pyramidal neurons through EphA7-mediated adhesion [19, 20]. Genome-wide association studies (GWAS) identify Satb2 as a locus associated with schizophrenia and cognitive abilities [21, 22]. Consistent with these genetic findings, mice with Satb2 inactivation in the cerebral cortex and hippocampus exhibit hyperactivity, social deficits and cognitive impairments [23–25]. Although Satb2 is expressed across all neocortical layers, its specific role in layer 6b is unknown.

Here we explored the role of Satb2 in the development of layer 6b in mice. In the absence of Satb2, expression of layer 6b-specific genes displayed opposite alterations. The expression of Ctgf, Cplx3, Trh and Tnmd were significantly reduced, whereas that of Nr4a2 was dramatically increased. To examine the involvement of the increased Nr4a2 expression, we generated Nr4a2 CKO mice that showed increased expression of Trh and Tnmd in layer 6b. When both Satb2 and Nr4a2 were inactivated, changes in these genes in layer 6b were very similar to those observed in mice with the deletion of Satb2 only. These results indicate that Satb2 and Nr4a2 are involved in the differentiation of layer 6b neurons possibly through different pathways.

## Results

### Satb2 is expressed in layer 6b neurons

**Fig. 1:**
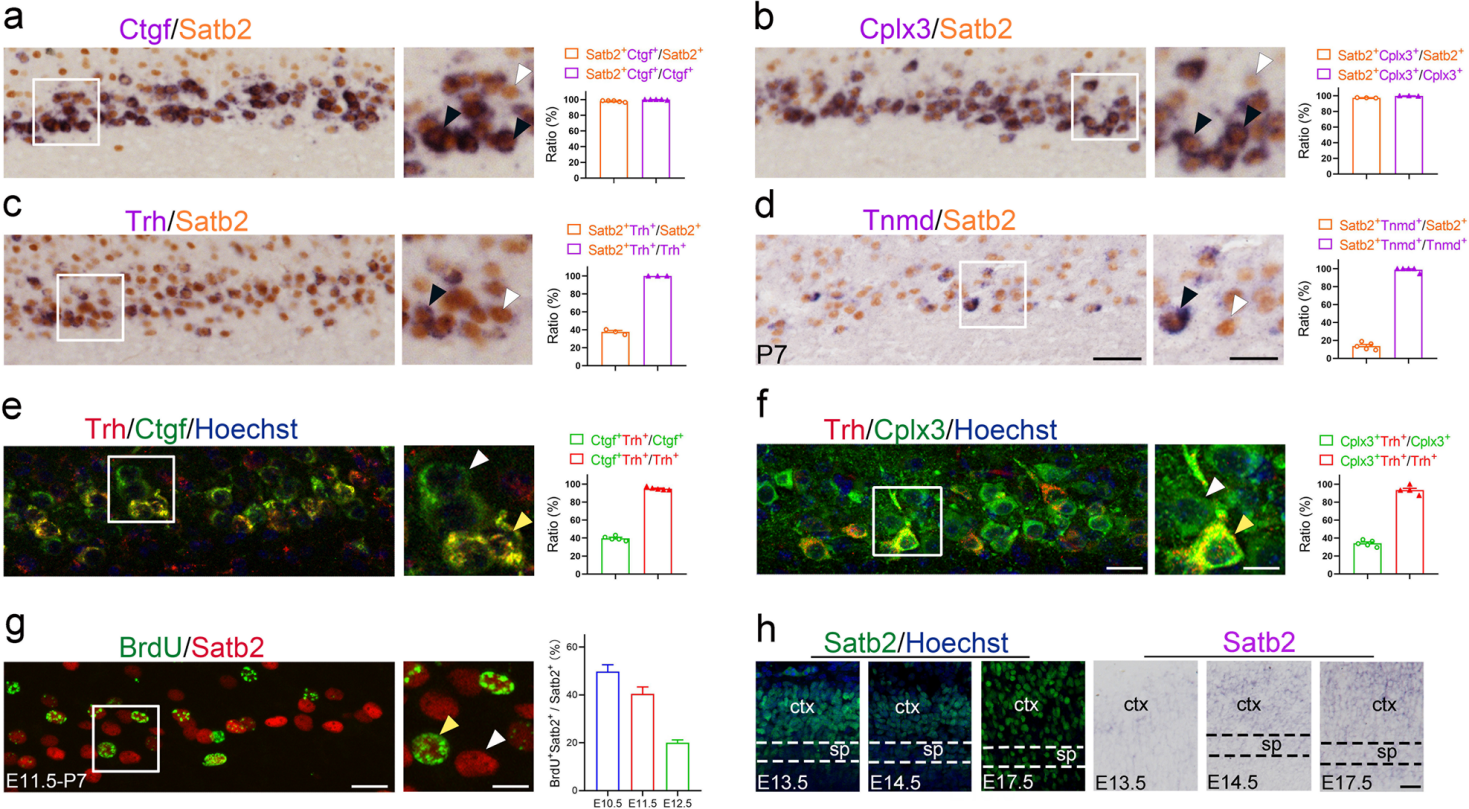
Cellular localization of Satb2 in layer 6b. **a**, **b** Colocalization of Satb2 with Ctgf and Cplx3 in layer 6b at P7. Satb2 (brown in the nucleus) was stained with an anti-Satb2 antibody, and Ctgf and Cplx3 (purple in the cytoplasm) were revealed by in situ hybridization. Cell counts show that the majority of Satb2^+^ neurons are co-labeled with Ctgf or Cplx3, and vice versa. **c**, **d** Colocalization of Satb2 with Trh and Tnmd in layer 6b at P7. Satb2 (brown in the nucleus) was stained with an anti-Satb2 antibody, and Trh and Tnmd (purple in the cytoplasm) were revealed by in situ hybridization. Cell counts show that Satb2^+^ neurons are present in these two subpopulations of layer 6b neurons. **e**, **f** Double immunostaining of Trh (red, **e**, **f**) with Ctgf (green, **e**) and Cplx3 (green, **f**) in layer 6b, showing that Trh represents a subpopulation of layer 6b neurons at P7. **g** Colocalization of BrdU and Satb2 in layer 6b of P7 mice with a single pulse of BrdU injection in pregnant mice at E11.5. Cell counts show that 40% of the Satb2^+^ neurons in layer 6b are born at this stage. **h** Dynamic expression of Satb2 protein and mRNA in the developing cortex at indicated timepoints. Both the protein and mRNA are clearly detected in the cortical plate including the subplate (Ctx) at E13.5, E14.5 and E17.5. Ctx cortex, sp subplate. Scale bars = 50 μm in (**a**–**d**), 25 μm in enlarged figures in (**a**–**d**), 20 μm in (**e**, **f**), 10 μm in enlarged figures in (**e**, **f**), 20 μm in (**g**), 10 μm in enlarged figure in (**g**), 20 μm in (**h**).

We previously reported that Satb2 is expressed in excitatory neurons across all cortical layers [26]. Here we focused on the cellular characterization of Satb2 in layer 6b. Ctgf and Cplx3 are exclusively expressed in layer 6b [27], and therefore were used as layer 6b neuron markers to examine cellular distribution of Satb2. We found that the vast majority of Ctgf^+^ (99.8%) and Cplx3^+^ (99.8%) neurons were immunostained with Satb2, which corresponded to 97.9% and 97.5% of Satb2^+^ neurons, respectively (Fig. 1a, b). Until now, fewer studies have focused on characterization of neuronal types in layer 6b. However, thanks to single-cell RNA sequencing, more genes were identified in layer 6b [1]. It has been shown that expression of Trh (thyrotropin-releasing hormone) and Tnmd (tenomodulin) is restricted to layer 6b in the cerebral cortex (Allen Brain Atlas), and our data showed that Satb2 was expressed in nearly all Trh^+^ and Tnmd^+^ neurons (Fig. 1c, d). Satb2/Trh-double-labeled neurons occupied about 37.6% of Satb2^+^ neurons, and Satb2/Tnmd-double-labeled neurons occupied approximately 13.7% of Satb2^+^ neurons (Fig. 1c, d), suggesting that Trh and Tnmd represent a subpopulation of layer 6b neurons. To confirm this, colocalization of Trh with Ctgf ad Cplx3 was examined. It showed that almost all Trh^+^ neurons were also immunoreactive for Ctgf (Fig. 1e) and Cplx3 (Fig. 1f), and the double-labeled neurons were present in about 39.7% and 34.1% of Ctgf- and Cplx3-expressing neurons, respectively (Fig. 1e, f). Taken together, Satb2 is expressed in all layer 6b neurons and Trh/Tnmd defines subclasses of layer 6b neurons.

Layer 6b neurons are located in the subplate and are the first wave of neurons born in corticogenesis [28]. To determine the birthdate of Satb2-expressing neurons in layer 6b, a single pulse of BrdU was injected at E10.5, E11.5 or E12.5 and the proportions of BrdU^+^/Satb2^+^ neurons in the total of Satb2^+^ cells in layer 6b were about 49.9%, 40.4% and 20.0%, respectively (Figs. 1g and S1) showing Satb2^+^ neurons in layer 6b are predominately born during E10.5-E12.5. Dynamic expression of Satb2 protein and mRNA was also examined, and their expression were clearly detected in the cortical plate from E13.5 (Fig. 1h). Therefore, Satb2 expresses strongly and persistently in the layer 6b neurons from embryonic stage.

### Satb2 is required for the differentiation of layer 6b

**Fig. 2:**
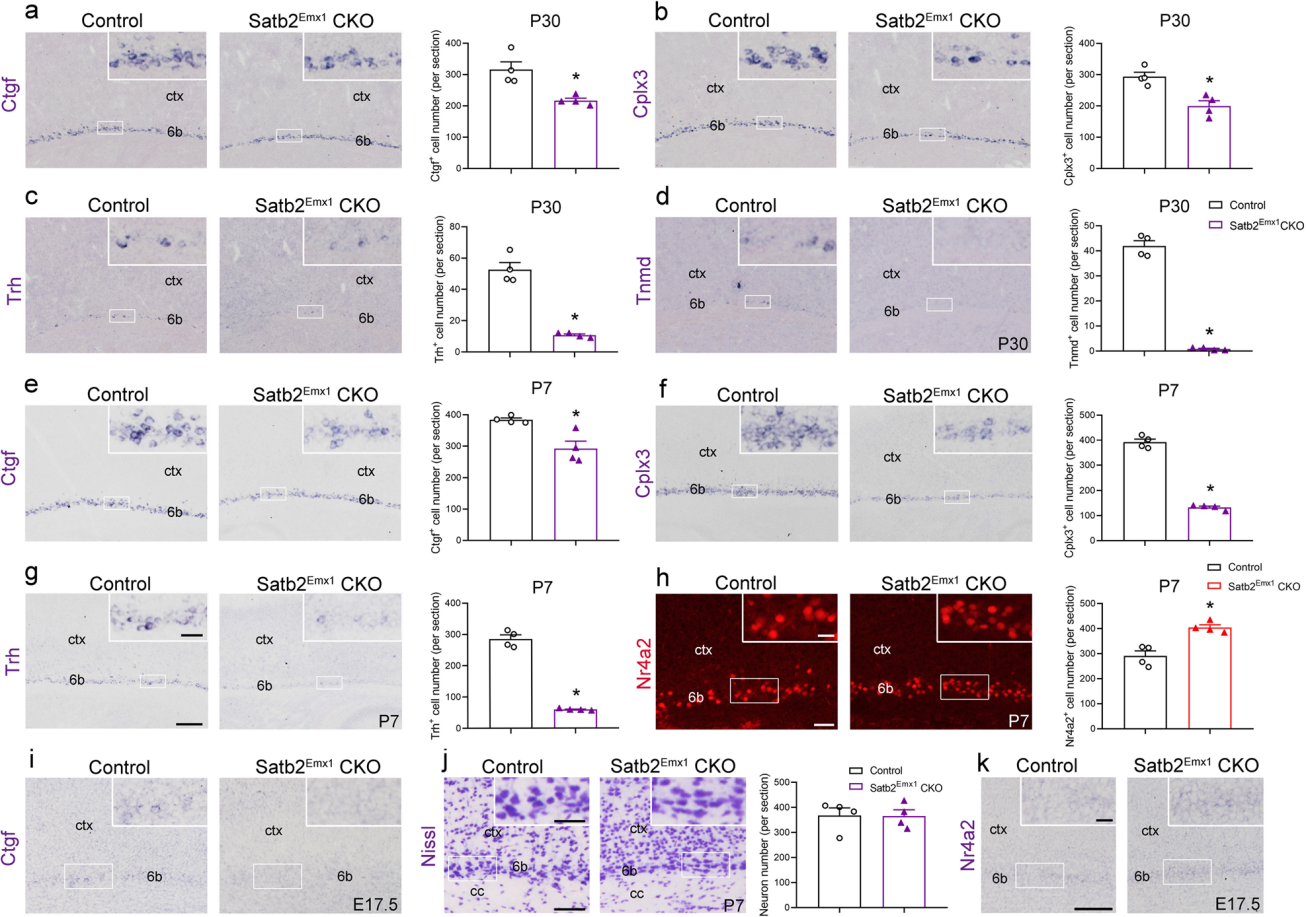
Defective differentiation of layer 6b neurons in Satb2^Emx1^ CKO mice. **a**–**g** In situ hybridization shows reduced Ctgf (**a**), Cplx3 (**b**), Trh (**c**) and Tnmd (**d**) positive cell number in layer 6b of Satb2^Emx1^ CKO mice compared with controls at P30. In situ hybridization shows reduced Ctgf (**e**), Cplx3 (**f**) and Trh (**g**) positive cell number in layer 6b of Satb2^Emx1^ CKO mice compared with controls at P7. **h** Immunohistochemistry shows increased Nr4a2^+^ cell number in layer 6b of Satb2^Emx1^ CKO mice compared with controls at P7. **i** In situ hybridization shows weak signals for Ctgf mRNA in the subplate of control embryos but undetectable in Satb2^Emx1^ CKO mice at E17.5. **j** Nissl staining shows no obvious difference in cellular architecture of layer 6b between controls and Satb2^Emx1^ CKO mice at P7. **k** In situ hybridization shows increased Nr4a2^+^ cell number in the subplate of Satb2^Emx1^ CKO mice compared with controls at E17.5. Ctx cortex, sp subplate. *n* = 4 for each group in **a**–**h** and **j** for statistics, *P < 0.05. Scale bars = 100 μm in (**a**–**g**), 20 μm in inserts in (**a**–**g**), 50 μm in (**h**), 20 μm in inserts in (**h**), 100 μm in (**i**, **k**), 20– μm in inserts in (**i**, **k**), 50 μm in (**j**), 25 μm in inserts in (**j**).

To investigate the role of Satb2 in the development of layer 6b, we used Satb2^Emx1^ CKO (Emx1-Cre:Satb2^flox/flox^) mice in which Satb2 is deleted in the cerebral cortex and hippocampus at embryonic stage [23]. The expression of layer 6b-specific genes was examined in Satb2^Emx1^ CKO mice at P30. The number of Ctgf^+^ neurons was significantly reduced by about 30% in Satb2^Emx1^ CKO mice compared to controls (Fig. 2a). The number of Cplx3^+^ neurons showed a similar decrease in Satb2^Emx1^ CKO mice as Ctgf^+^ neurons (Fig. 2b). In addition, the expression of layer 6b subclass genes, Trh and Tnmd was also drastically decreased in Satb2^Emx1^ CKO mice relative to controls at P30 (Fig. 2c, d). In addition, the decreased expression of these genes was also evident at P7 (Fig. 2e–g). Next, we moved to examine their expressions in the cortex at embryonic stage. Among them, only Ctgf was weakly detected in the subplate of control embryos at E17.5, and it was hardly detected in Satb2^Emx1^ CKO embryos at this stage (Fig. 2i), showing the initial expression of layer 6b-specific gene is impaired in the absence of Satb2. It should be noted that cellular architecture of layer 6b was maintained as shown by the presence of relatively large Nissl-stained neurons loosely distributed along the white matter in Satb2^Emx1^ CKO mice (Fig. 2j).

Transcription factor Nr4a2 also shows layer 6b-restricted expression pattern in the neocortex [4]. Previously we have also shown that Satb2 determines the patterning of retrosplenial cortex by repressing Nr4a2 [18]. Therefore, we set out to explore whether the expression of Nr4a2 was altered in Satb2^Emx1^ CKO mice, and found that the number of Nr4a2^+^ cells was dramatically increased in layer 6b in Satb2^Emx1^ CKO mice compared to control mice at P7 (Fig. 2h). The increased expression of Nr4a2 could be detected as early at E17.5 (Fig. 2k). Taken together, the abnormal gene expression of layer 6b in Satb2^Emx1^ CKO mice indicates that Satb2 is required for the normal differentiation of layer 6b neurons.

### Nr4a2 is expressed in a subclass of layer 6b neurons

**Fig. 3:**
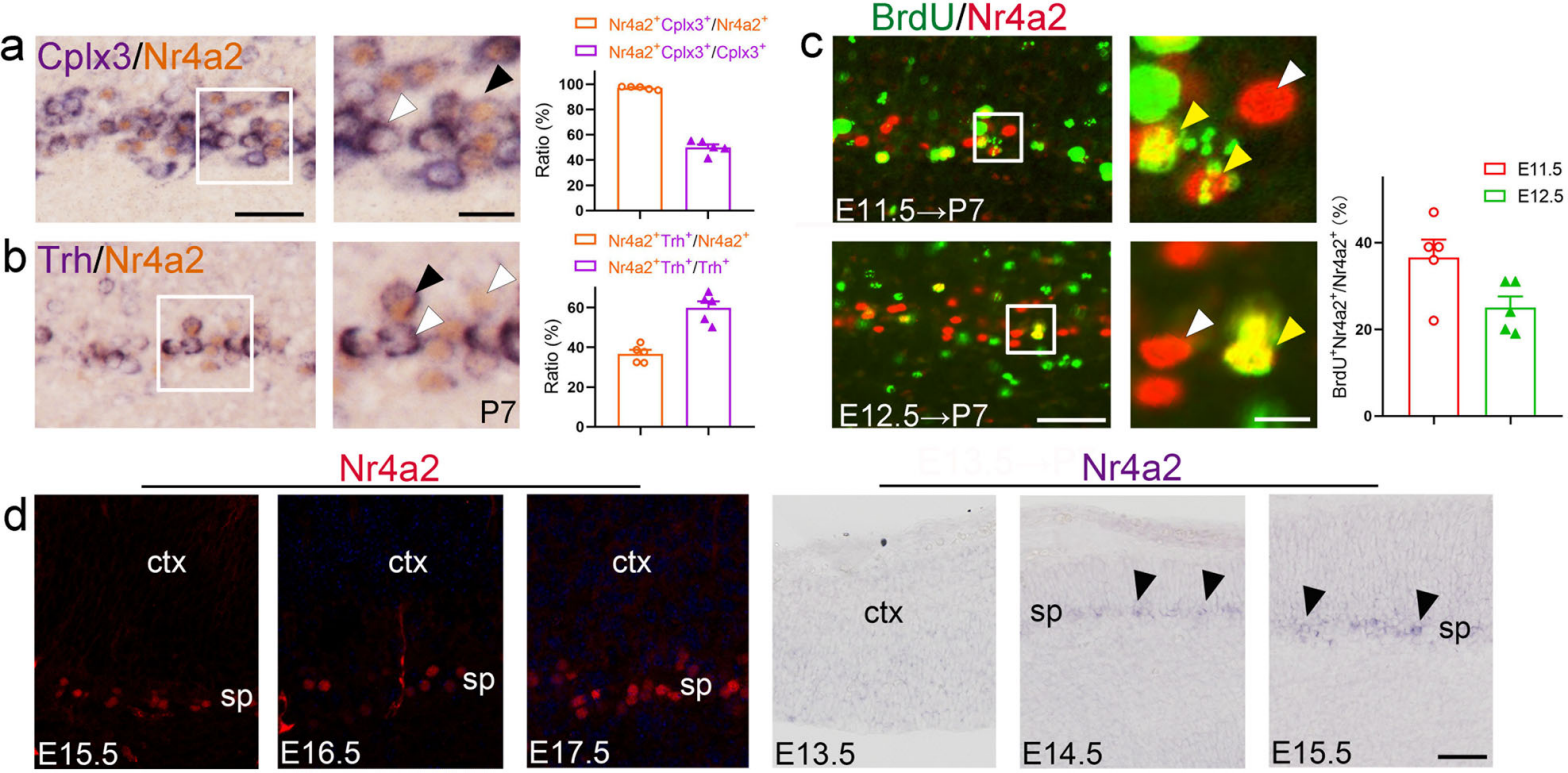
Cellular localization of Nr4a2 in layer 6b. **a**, **b** Colocalization of Nr4a2 with Cplx3 (**a**) and Trh (**b**) in layer 6b at P7. Nr4a2 (brown in nuclei) was revealed by immunohistochemistry, and Cplx3 and Trh (purple in cytoplasm) were revealed by in situ hybridization. Cell counts show that Nr4a2 is present in these two subpopulations of layer 6b neurons. Black arrowheads indicate double-labeled neurons and white arrowheads indicate single-labeled neurons. **c** Colocalization of BrdU (green) and Nr4a2 (red) in layer 6b of P7 mice with a single pulse of BrdU injection in pregnant mice at E11.5 and E12.5. Yellow arrowheads indicate double-labeled neurons and white arrowheads indicate single-labeled neurons. **d** Dynamic expression of Nr4a2 protein and mRNA in the developing cortex at indicated time points. Nr4a2 protein is clearly detected in the subplate at E15.5 and its mRNA is clearly detected in the subplate from E14.5. Black arrowheads indicate the subplate. Ctx cortex, sp subplate. Scale bars = 50 μm in (**a**, **b**), 20 μm in enlarged figures in (**a**, **b**), 50 μm in (**c**), 10 μm in enlarged figures in (**c**), 50 μm in (**d**).

The increase of Nr4a2^+^ neurons in Satb2^Emx1^ CKO mice prompted us to explore if Nr4a2 is also involved in the development of layer 6b and examine possible contribution of the upregulation of Nr4a2 in Satb2-controled layer 6b development. Firstly, the colocalization of Nr4a2 with the layer 6b-specific markers (i.e., Cplx3) was examined. Over 90% of Nr4a2^+^ neurons were found to be immunoreactive for Cplx3, while about 50.0% of Cplx3^+^ neurons were positive for Nr4a2 at P7 (Fig. 3a). In addition, double labeling of Nr4a2 and Trh showed that approximately 36.7% of Nr4a2^+^ neurons were Trh positive and 59.8% of Trh^+^ neurons were immunostained with Nr4a2 (Fig. 3b). These data indicate that Nr4a2 is expressed in a subclass of layer 6b neurons and may represent a larger subpopulation than the one expressing Trh.

The birthdates of Nr4a2^+^ neurons were then examined using BrdU-pulse labeling. Pregnant mice at different stages of embryonic development (E11.5 and E12.5) were injected with a single pulse of BrdU and the pups were sacrificed for analysis at P7. Colocalization of BrdU and Nr4a2 revealed that most of the BrdU^+^/Nr4a2^+^ neurons were observed in layer 6b between E11.5 and E12.5 (Fig. 3c), which is similar to that of Satb2^+^ neurons in layer 6b. Finally, the expression of Nr4a2 protein and mRNA in the subplate or layer 6b was examined during embryonic cortical development. The expression of Nr4a2 protein could be clearly detected at E15.5 in the subplate, while the transcription of Nr4a2 was clearly detected from E14.5 (Fig. 3d). Taken together, Nr4a2 is expressed exclusively in cortical layer 6b.

### Nr4a2 is also required for the differentiation of layer 6b neurons

**Fig. 4:**
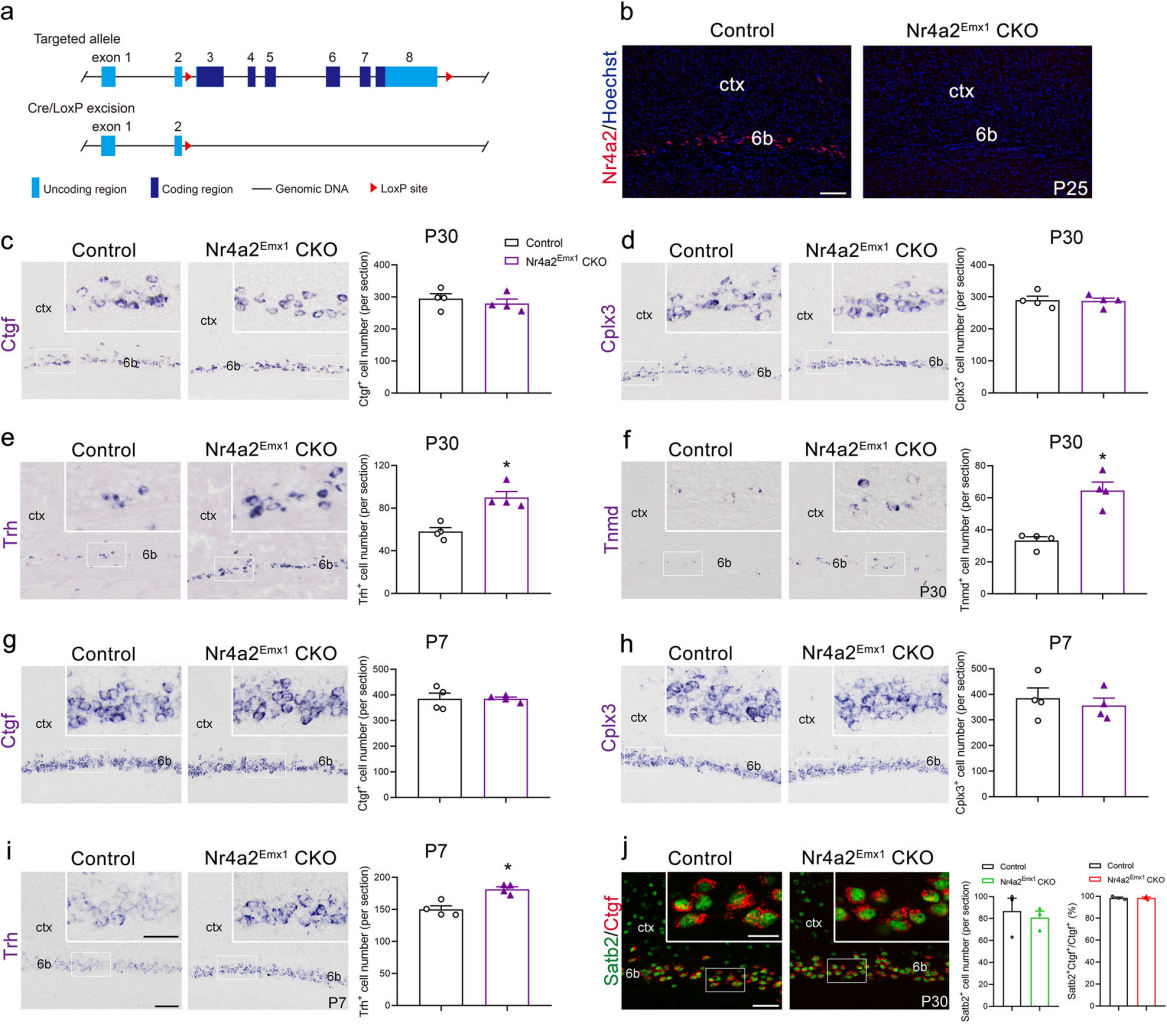
Altered gene expression in the layer 6b of Nr4a2^Emx1^ CKO mice. **a** The strategy for generating a mouse line with Cre-dependent Nr4a2 inactivation. **b** Loss of Nr4a2 expression in layer 6b of Nr4a2^Emx1^ CKO mice compared with controls. **c**–**i** The cell number of Ctgf (**c**) and Cplx3 (**d**) is comparable between Nr4a2^Emx1^ CKO and control mice, while the number of Trh^+^ (**e**) and Tnmd^+^ (**f**) neurons is increased in Nr4a2^Emx1^ CKO mice compared with controls at P30. The cell number of Ctgf (**g**) and Cplx3 (**h**) is comparable between Nr4a2^Emx1^ CKO and control mice, and the number of Trh^+^ (**i**) neurons is increased in Nr4a2^Emx1^ CKO mice compared with controls at P7. **j** There is no change in the number of Satb2^+^ neurons or the ratio of Satb2^+^Ctgf^+^/Ctgf^+^ neurons between controls and Nr4a2^Emx1^ CKO mice at P30. Ctx cortex. *n* = 4 for each group in **c**–**j** for statistics, *P < 0.05. Scale bars = 100 μm in (**b**), 100 μm in (**c**–**i**), 50 μm in inserts in (**c**–**i**), 50 μm in (**j**), 20 μm in inserts in (**j**).

Nr4a2 in layer 6b was upregulated in Satb2^Emx1^ CKO mice (Fig. 2h, k) suggesting that Satb2 may function through Nr4a2 in regulating the development of layer 6b. To test this, we generated a floxed Nr4a2 mouse line in which exons 3–8 were flanked by loxp sites (Fig. 4a). Floxed Nr4a2 mice were then crossed with the Emx1-Cre line [29] to delete exons 3–8 by recombination, resulting in Nr4a2^Emx1^ CKO (Emx1-Cre:Nr4a2^flox/flox^) mice (Fig. 4a). Immunostaining of Nr4a2 showed the absence of Nr4a2 immunoreactivity in the cerebral cortex of Nr4a2^Emx1^ CKO mice (Fig. 4b).

Next, the expression of the layer 6b-specific genes was examined in Nr4a2^Emx1^ CKO mice. Unexpectedly, the number of Ctgf^+^ and Cplx3^+^ cells was comparable between the two genotypes, but more Trh^+^ and Tnmd^+^ neurons were detected in Nr4a2^Emx1^ CKO mice comparing to that in control mice at P7 and P30 (Fig. 4c–i). Because Nr4a2 was up-regulated in Satb2^Emx1^ CKO mice, we were promoted to examined whether Satb2 expression is altered in Nr4a2^Emx1^ CKO mice. Double immunostaining of Satb2 and Ctgf showed no differences in the number of Satb2^+^ neurons or ratio of Satb2^+^Ctgf^+^/Ctgf^+^ neurons between control and Nr4a2^Emx1^ CKO mice (Fig. 4j). These results indicated that Nr4a2 is also involved in the differentiation of layer 6b neurons. Distinct from the phenotype in Satb2^Emx1^ CKO mice, the deletion of Nr4a2 leads to increase rather than deceased expression of the subpopulation-specific genes, Trh and Tnmd in layer 6b.

### Satb2 and Nr4a2 are involved in the differentiation of layer 6b neurons possibly through different pathways

**Fig. 5:**
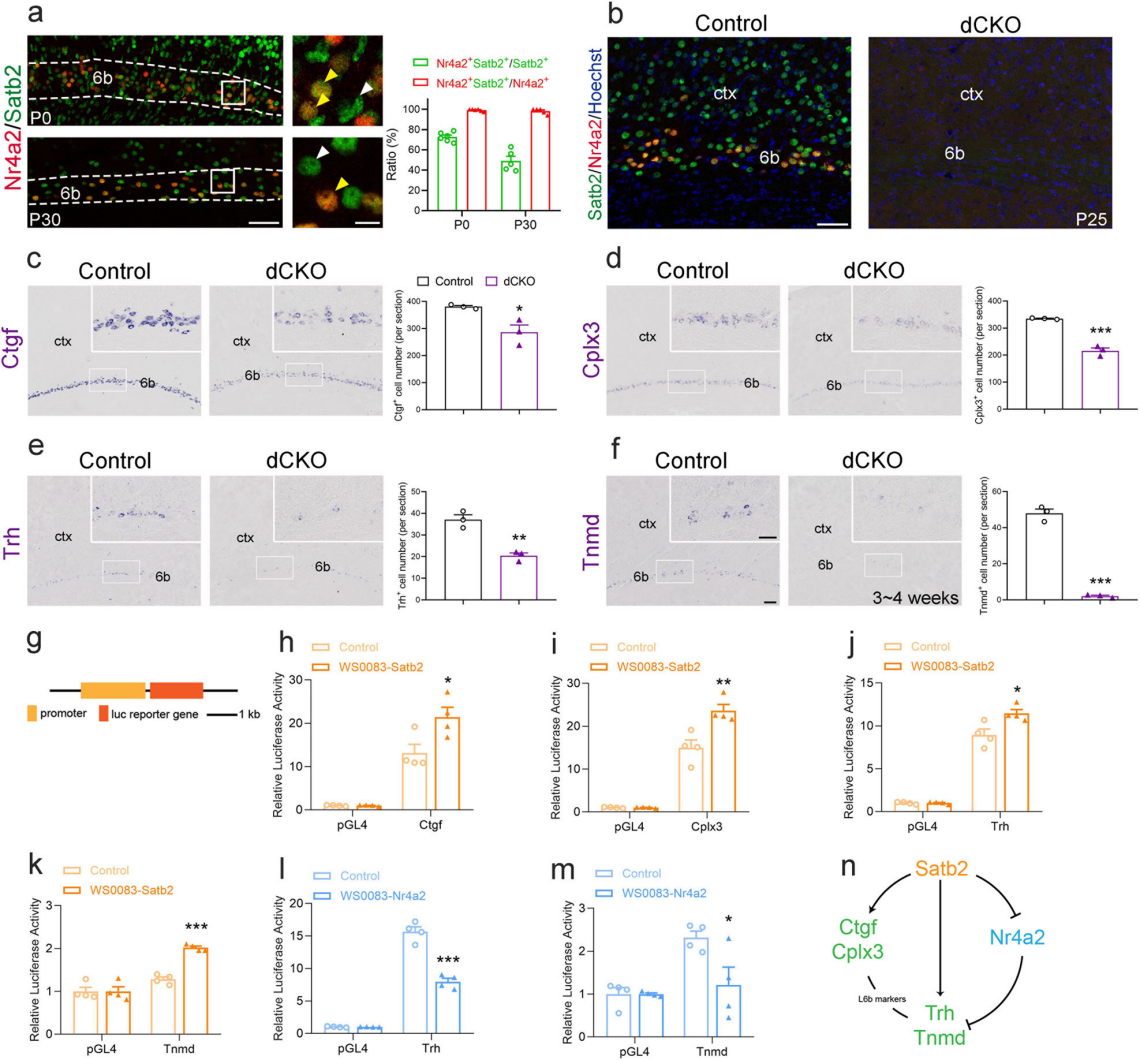
Satb2/Nr4a2 dCKO mice phenocopy what observed in Satb2^Emx1^ single CKO mice. **a** Double immunostaining of Nr4a2 (red) with Satb2 (green) in layer 6b at P0 and P30. Cell counts show that about half Satb2^+^ neurons are co-labeled with Nr4a2 and almost all Nr4a2^+^ neurons are positive for Satb2 in layer 6b of control mice at P0 and P30. Yellow arrowheads indicate Satb2^+^/Nr4a2^+^ colabeled neurons and white arrowheads indicate single-labeled neurons. **b** Deletion of Satb2 and Nr4a2 is shown by immunostaining in the cortex of Satb2/Nr4a2 dCKO mice compared with controls at P25. **c**–**f** The number of Ctgf^+^ (**c**), Cplx3^+^ (**d**), Trh^+^ (**e**) and Tnmd^+^ (**f**) neurons is significantly reduced in the layer 6b of Satb2/Nr4a2 dCKO compared to controls. **g** Diagram of the construction of luciferase report gene plasmids. **h**–**k** Luciferase reporter assay shows that the transcriptional activities of the Ctgf, Cplx3, Trh, and Tnmd are significantly increased in the presence of Satb2 overexpression. **l**, **m** The transcriptional repression of Trh and Tnmd is detected in the presence of Nr4a2 overexpression. **n** Proposed model: Satb2 promotes the transcription of Ctgf, Cplx3, Trh, and Tnmd, while Nr4a2 suppresses the transcription of Trh and Tnmd during the differentiation of layer 6b neurons. dCKO, Satb2/Nr4a2 double CKO mice; Ctx, cortex. *n* = 3 for each group in **c**–**f** for statistics, *n* = 4 for each group in **h**–**m** for statistics, *P < 0.05, **P < 0.01,***P < 0.001. Scale bars = 50 μm in (**a**), 10 μm in enlarged figures in (**a**), 50 μm in (**b**), 100 μm in (**c**–**f**), 50 μm in inserts in (**c**–**f**).

Having found that the up-regulation of Nr4a2 in Satb2^Emx1^ CKO mice and opposite alterations of layer 6b-specific gene expression in Nr4a2^Emx1^ CKO mice, we speculated that Nr4a2 may function as a downstream gene in the regulation of layer 6b development. We first examined colocalization of Nr4a2 with Satb2, and double immunostaining of Nr4a2 and Satb2 showed that almost all Nr4a2^+^ neurons were Satb2 positive at P7 and P30, which corresponded to approximately 73.1% or 49.0% of Satb2^+^ neurons at P0 and P30, respectively (Fig. 5a). Then we generated Satb2/Nr4a2 double CKO (Emx1-Cre:Satb2^flox/flox^:Nr4a2 ^flox/flox^) mice (Fig. 5b). Our data showed that the number of Ctgf^+^, Cplx3^+^, Trh^+^, and Tnmd^+^ neurons was all significantly reduced in the double CKO mice compared to control mice (Fig. 5c–f), and critically cell counts showed the reductions in double CKO mice were similar to those observed in Satb2^Emx1^ CKO mice (Fig. 2). These results demonstrate that the loss of Satb2 overrode Nr4a2 in regulating the development of layer 6b, although Nr4a2 is upregulated in the absence of Satb2.

As transcription factors, Satb2 and Nr4a2 may directly bind to promoters or enhancers of downstream genes to regulate their transcriptions. To test whether and how Satb2/Nr4a2 regulates the transcription of these genes in vitro, we performed a dual luciferase assay, in which certain DNA motifs were inserted into the promoter region of the luciferase gene to measure its transcription activity in the presence of Satb2 or Nr4a2 overexpression (Fig. 5g). As expected, the motifs in Ctgf, Cplx3, Trh, and Tnmd genomes significantly increased the luciferase transcription in the presence of Satb2 (Fig. 5h–k), while the motifs in Trh and Tnmd genomes dramatically reduced the transcription in the presence of Nr4a2 (Fig. 5l, m). These results indicate that Satb2 promotes the transcription of Ctgf, Cplx3, Trh and Tnmd, while Nr4a2 inhibits the transcription of Trh and Tnmd (Fig. 5n).

### Cell autonomous role of Satb2 in the regulation of layer 6b neuron differentiation

**Fig. 6:**
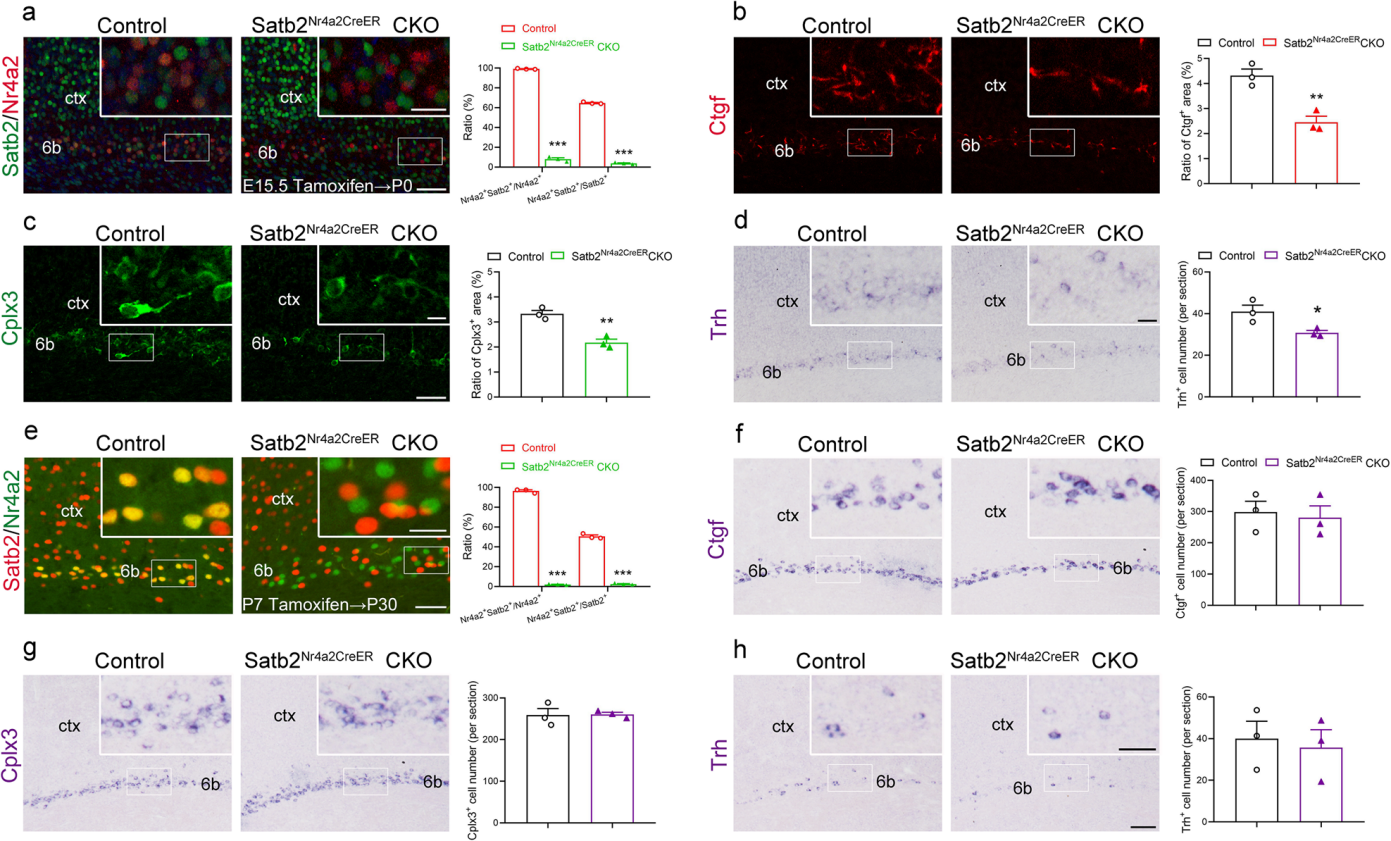
Cell autonomous role of Satb2 in the differentiation of layer 6b neurons. **a** Satb2 is deleted in Nr4a2^+^ cells in layer 6b in Satb2^Nr4a2CreER^ CKO mice at P0 with tamoxifen injection at E15.5. **b**–**d** The expression of Ctgf (**b**), Cplx3 (**c**) or Trh (**d**) is significantly reduced in Satb2^Nr4a2CreER^ CKO mice compared to control mice at P0. The quantitation of Ctgf^+^, Cplx3^+^ and Trh^+^ cell counts is shown in (**b**–**d**). **e** Satb2 is deleted in Nr4a2^+^ cells in layer 6b in Satb2^Nr4a2CreER^ CKO mice with tamoxifen injection at P7. **f**–**h** The number of Ctgf^+^ (**f**), Cplx3^+^ (**g**) and Trh^+^ (**h**) cells is comparable between controls and Satb2^Nr4a2CreER^ CKO mice at P30. *n* = 3 for each group in a-h for statistics, *P < 0.05, **P < 0.01,***P < 0.001. Scale bars = 50 μm in (**a**), 20 μm in inserts in (**a**), 100 μm in (**b**, **c**), 20 μm in inserts in (**b**, **c**), 100 μm in (**d**), 20 μm in inserts in (**d**), 50 μm in (**e**), 20 μm in inserts in (**e**), 100 μm in (**f**–**h**), 50 μm in inserts in (**f**–**h**).

As mentioned above, Satb2 is a fate determination gene for callosal projection neurons in the superficial cerebral cortex and also involved in the patterning of transitional cortex (i.e., retrosplenial cortex) that located between the neocortex and hippocampal formation [16, 18]. The drastic alterations in the cerebral cortex of Satb2^Emx1^ CKO mice may lead to secondary changes in layer 6b. To address this question, we set out to inactivate Satb2 by selective deletion of Satb2 in layer 6b neurons. Nr4a2-Cre^ER^ mouse line was generated and Satb2^Nr4a2CreER^ CKO mice were obtained. When tamoxifen was injected into Satb2^Nr4a2CreER^ CKO mice at E15.5 (Figs. 1h, 3d), few of Nr4a2^+^ neurons were immunostained with Satb2 in layer 6b (Fig. 6a), showing deletion of Satb2 in Nr4a2^+^ population. Consistent with the data from Satb2^Emx1^ CKO mice (Fig. 2), the expression of Ctgf, Cplx3 and Trh was significantly reduced in Satb2^Nr4a2CreER^ CKO mice compared to those in control mice at P0 (Fig. 6b-d). When tamoxifen was administered at P7, Satb2 was also inactivated in Nr4a2^+^ neurons (Fig. 6e), but the number of Ctgf^+^, Cplx3^+^ and Trh^+^ cells was comparable between control and Satb2^Nr4a2CreER^ CKO mice at P30 (Fig. 6f–h). These results indicate Satb2 is involved in the differentiation of layer 6b in a cell-autonomous manner at embryonic stage, while dispensable for the maintenance of the gene expressions in layer 6b during postnatal development.

## Discussion

In this study, we examined the role of Satb2 in the development of layer 6b by selective deletion of it with the help of Emx1-driven Cre line. In the absence of Satb2, the differentiation of layer 6b neurons is affected as shown by the altered gene expression. Because Nr4a2 expression is up-regulated, and we generated Nr4a2^Emx1^ CKO mice in which the opposite gene expression is observed. However, when both of them are inactivated, double CKO mice show similar changes to those in Satb2 single CKO mice. Our results provide evidence showing that both Satb2 and Nr4a2 are required for the differentiation of layer 6b neurons and the up-regulation of Nr4a2 is not sufficient to compensate the loss of Sab2-indued defective differentiation of layer 6b.

Layer 6b is derived from the subplate during embryonic development and is considered as a remnant of the subplate in adult brain [2]. Neurons in layer 6b are the earliest-generated neurons in the cerebral cortex, and recent single-cell RNA sequencing (scRNA-seq) analysis confirms that layer 6b is a quite distinct layer from layer 6a in terms of transcriptome and subclasses of projection neurons [1]. Neurons in layer 6b are morphologically divided into five distinct clusters of excitatory neurons [30, 31], and also classified according to the gene expression profiles [1]. Our data confirmed that Ctgf and Cplx3 represent the whole population of layer 6b neurons, and Trh and Tnmd are expressed in the subpopulations of layer 6b neurons. Like these genes, Nr4a2 also shows a narrow-band distribution pattern in layer 6b, and presents in about 60% of Trh^+^ subpopulations. Our data support the idea that layer 6b neurons are molecularly heterogeneous, and these genes serve as unique markers for studying molecular alterations of layer 6b neurons in cortical development.

We report that two transcription factors, Satb2 and Nr4a2 have different functions in layer 6b development. In Satb2^Emx1^ CKO mice, the number of Ctgf^+^, Cplx3^+^, Trh^+^ and Tnmd^+^ neurons is decreased but that of Nr4a2^+^ neurons is increased. To examine possible contributions of the increased Nr4a2 in Satb2-controlled developmental process, we generated Nr4a2^Emx1^ CKO mice and found the decrease of Trh^+^ and Tnmd^+^ neurons with no change in Ctgf^+^ and Cplx3^+^ populations. Thus, Satb2 and Nr4a2 are required for the regulation of layer 6b neurons with distinct functions. In support of this, our i*n vitro* data demonstrate that Satb2 positively regulates the transcription of Ctgf, Cplx3, Trh and Tnmd, while Nr4a2 negatively regulates transcription of Trh and Tnmd.

We have previously shown that Satb2 binds to the MAR in the Nr4a2 genomic region and suppresses its expression in the regionalization of the retrosplenial cortex [18]. In this study, we observed a significant increase in Nr4a2^+^ neurons in layer 6b of Satb2^Emx1^ CKO mice, and therefore inferred that Nr4a2 might also serve as the downstream factor of Satb2 in layer 6b development as it does in the retrosplenial cortex. On the other hand, the altered gene expression in Satb2/Nr4a2-double CKO mice phenocopied what observed in Satb2 single CKO mice. One possible explanation is that the upregulation of Nr4a2 is not sufficient to compensate for the defective differentiation of layer 6b neurons caused by the loss of Satb2. This is very likely to be the case based on the fact that simultaneous misexpression of Nr4a2 and Ctip2 in Satb2-mutant neurons of retrosplenial cortex is capable of preventing neuronal fate change from the retrosplenial cortex to the subiculum [18]. An alternative possibility is that Satb2 may regulate the development of layer 6b neurons in an Nr4a2-independent way.

Satb2 is expressed in all cortical layers, and the laminated architecture is totally disrupted in Satb2 KO mice [16, 23]. This raises a question of the defective differentiation of layer 6b in the Satb2^Emx1^ CKO mice may be secondary but not caused by the deletion of Satb2 in layer 6b neurons. However, our Satb2^Nr4a2CreER^ CKO mice with selective deletion of Satb2 in Nr4a2^+^ neurons show similar alterations of layer 6b-specific genes, indicting the cell autonomous effect of Satb2 in the regulation of layer 6b neuron differentiation. It should be noted that when Satb2 was deleted postnatally, the expression of these genes remained unchanged, showing that Satb2 is dispensable for the maintenance of these gene expression during postnatal development.

In summary, our data indicate that Satb2 and Nr4a2 are required for the differentiation of layer 6b with different molecular mechanisms, and future study is needed to explore possible functional consequences caused by the deletion because layer 6b has been proposed to have multiple roles in higher brain functions as well as in psychiatric disorders [3, 5, 6, 8].

## Materials and methods

### Animals

Satb2^flox/flox^ mice were generated as previously described [18], and Nr4a2^flox/flox^ mice were generated with two LoxP sites flanking exons 3-8. Nr4a2-P2A-Cre^ERT2^-IRES-EGFP mice (Nr4a2-Cre^ER^ mice) were constructed with CRISPR/Cas9 techniques. To conditionally delete Satb2 gene in layer 6b, floxed Satb2 mice were crossed with Emx1-Cre mice [29] or Nr4a2-Cre^ER^ mice, and Satb2^Emx1^ CKO (Emx1-Cre:Satb2^flox/flox^) or Satb2^Nr4a2CreER^ CKO (Nr4a2-Cre^ER^:Satb2^flox/flox^) mice were obtained. The same method was used to generate Nr4a2^Emx1^ CKO (Emx1-Cre:Nr4a2^flox/flox^) mice. Other genotypes (i.e., Satb2^flox/flox^, Satb2^flox/+^, Nr4a2^flox/flox^ and Nr4a2^flox/+^) from the same litter were used as control mice. The day of the vaginal plug detection was recorded as embryonic day 0.5 (E0.5), and the day of birth was recorded as postnatal day 0 (P0). All mouse experiments were reviewed and approved by the Laboratory Animal Committee of Fudan University.

### Immunohistochemistry, BrdU labeling, and in situ hybridization

Anaesthetized mice were perfused with phosphate-buffered saline (PBS) and 4% paraformaldehyde (PFA), and then the brains were dissected out and placed in 4% PFA to fix overnight. After cryoprotection with 30% sucrose, the brains were sectioned by a cryostat (CM1950, Leica).

For immunohistochemistry, brain sections were treated with sodium citrate at 95 °C for 20 min or in microwave for 2 min followed by 95°C for 20 min, then incubated in primary antibodies: rabbit anti-Satb2 (1:300; ab92446, Abcam), mouse anti-Nr4a2 (1:300; ab41917, Abcam), goat anti-Ctgf (1:200; sc-14939, Santa Cruz), rabbit anti-Cplx3 (1:1000; 122302, Synaptic System), rabbit anti-Trh (1:500; PA5-57331, Thermo Fisher Scientific), and rat anti-BrdU (1:300; OBT0030G, Accurate Chemical and Scientific Corporation). Brain sections were incubated in primary antibodies at 4°C overnight and then with biotinylated or fluorophore-conjugated secondary antibodies at room temperature for 2 hours followed by incubation with streptavidin-Cy3 (1:1000; Jackson ImmunoResearch) if necessary. All brain sections were counterstained with Hoechst 33258 (1:1000; 94403, Sigma).

For BrdU (B9285, Sigma) labeling, pregnant mice were injected with 100 mg/kg body weight (i.p.), and brains were collected at postnatal day 7 (P7) of offspring mice. Brain sections were sequentially treated with sodium citrate at 95°C for 20 min, HCl (2 N) at 37 °C for 30 min, and sodium borate (0.1 M, pH 8.5) at room temperature for 10 min, then incubated with antibody as described above.

In situ hybridization (ISH) used digoxigenin UTP-labeled probes, performing as described previously [32]. Ctgf, Cplx3, Trh and Tnmd RNA probes were constructed according to the Allen Brain Atlas. For double labeling of ISH and immunostaining, brain sections were hybridized with RNA probes first. After visualization for mRNA, brain sections were incubated with rabbit anti-Satb2 antibody or mouse anti-Nr4a2 antibody overnight. The sections were incubated with biotinylated horse anti-rabbit antibody (1:500, Vector Laboratories) or horse anti-mouse antibody (1:500, Vector Laboratories) at room temperature for 2 hours. Then the sections were processed using ABC kit (1:200, Vector Laboratories) for 1 hour and were incubated with diaminobenzidine and H_2_O_2_ (3%) to visualize immunoreactivity.

### Luciferase reporter assay

HEK293T, N2A or DAOY cell lines were cultured in 48-well plates, and then transfected by Lipofectamine 3000 Reagent (Thermo Fisher Scientific). The N2A cell line was employed for Ctgf, Cplx3, and Trh, while DAOY and 293T cell lines were used for Tnmd. Cells in each well were transfected with 5 ng pGL4.73, 120 ng WS0083-Satb2/WS0083-Nr4a2 or empty construct, together with 120 ng reporter vector (Ctgf-luc, Cplx3-luc, Trh-luc, or Tnmd-luc). Promoterless pGL4 vectors were used to assess the binding of Satb2 to the target genes and Nr4a2 to Trh, whereas pGL vectors with a weak promoter were utilized to evaluate the binding of Nr4a2 to Tnmd. Each experiment included 3–4 replicate wells as technical replicates, with luciferase activity assayed in lysates from transfected cells of the same batch under consistent conditions. The primer sequences for the above gene promoters were as follows: Ctgf-F (CACTCATTCCACCTTTCTGACC), Ctgf-R (GAGTGGATCTGGCTGAGTCTTC), Cplx3-F (CCATCTGGCTGGCTTCTAGGAT), Cplx3-R (CACTGCTGGCTACTGCTGACA), Trh-F (TTCTTCCAGTACCCACCTT), Trh-R (AATCCAGAGTCGACTGCGC), Tnmd-F (ATATGAAGGGATGGGGAG), Tnmd-R (GTATATAACTTCTGTGCACAG). Twenty-four hours later, the cells were harvested, the luciferase assay was performed by Dual-Luciferase Reporter Assay System Kit (E1910, Promega).

### Statistical analysis

For cell count in layer 6b or subplate, each brain was cut into coronal sections. Counts were started and averaged after the first appearance of the hippocampus along the rostrocaudal axis in three adjacent sections in one hemisphere. Images were acquired using Nikon microscope (DS-Ri2) and Olympus confocal microscope (FV3000). GraphPad Prism (8.0.1) software was used for statistical analysis. Nonparametric test or Two-tailed Student’s *t* test were used for comparisons. All results were shown as means ± SEM. Statistical significance was indicated as *P <0.05, **P <0.01, ***P <0.001, and no significance (ns).

## Supplementary information

Supplemental Figure

## Data Availability

All data generated or analyzed during this study are included in this published article.
