# Supplementary material for: Satb2 and Nr4a2 are required for the differentiation of cortical layer 6b
Source: Cell Death Discov. 2025 Mar 31;11:126. doi: 10.1038/s41420-025-02402-2 (PMC11958660; doi:10.1038/s41420-025-02402-2)
Supplement: Supplementary file 1 — Supplemental Figure [file 41420_2025_2402_MOESM1_ESM.docx]

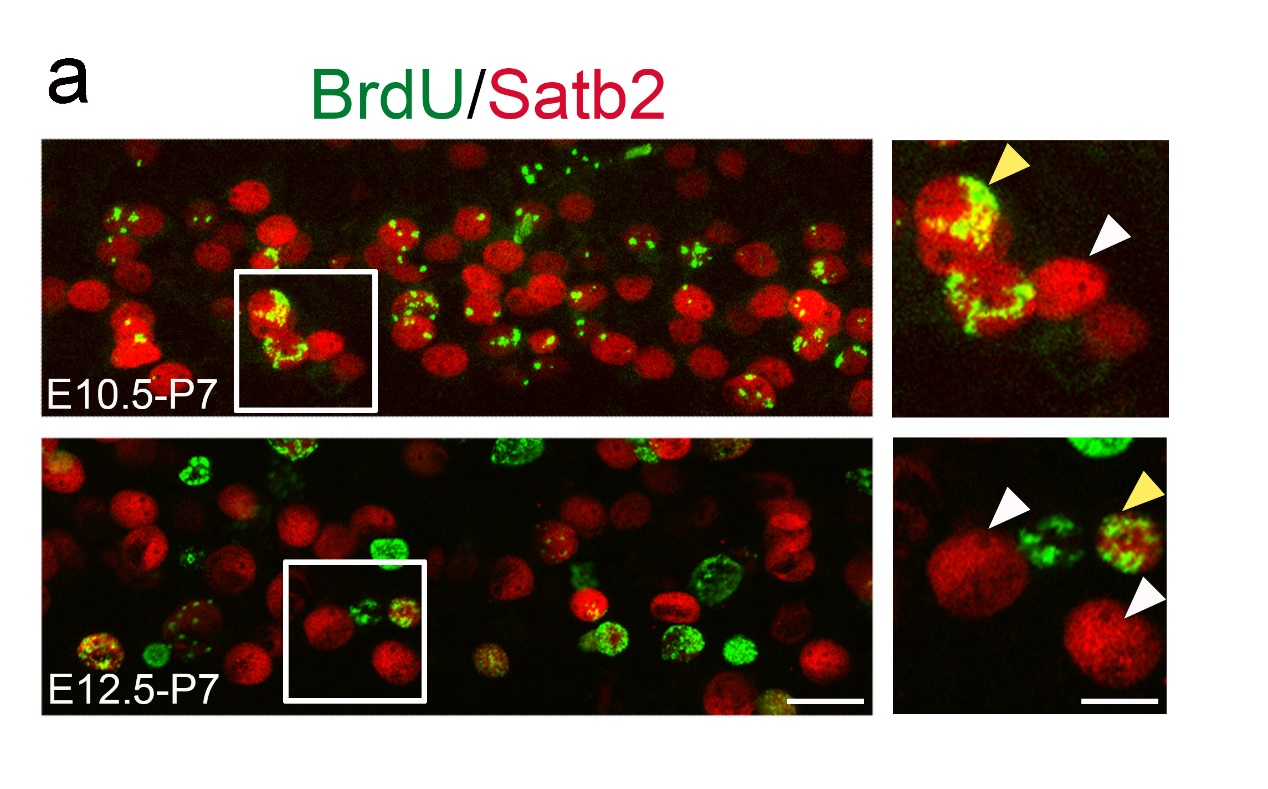


**Figure S1. Generation of Satb2^+^ neurons in layer 6b.**

(a) Colocalization of BrdU and Satb2 in layer 6b of P7 mice with a single pulse of BrdU injection in pregnant mice at E10.5 and E12.5.

Scale bars = 20 μm in a, 10 μm in enlarged figures in a.
